# β-Arrestin1 Reduces Oxidative Stress via Nrf2 Activation in the Rostral Ventrolateral Medulla in Hypertension

**DOI:** 10.3389/fnins.2021.657825

**Published:** 2021-04-07

**Authors:** Xing Tan, Pei-Lei Jiao, Jia-Cen Sun, Wen Wang, Peng Ye, Yang-Kai Wang, Yue-Qi Leng, Wei-Zhong Wang

**Affiliations:** ^1^Department of Marine Biomedicine and Polar Medicine, Naval Medical Center, Naval Medical University (Second Military Medical University), Shanghai, China; ^2^Department of Orthopedics, The 962th Hospital of People’s Liberation Army, Harbin, China

**Keywords:** hypertension, rostral ventrolateral medulla, β-arrestin1, oxidative stress, Nrf2

## Abstract

Oxidative stress in the rostral ventrolateral medulla (RVLM), a key region for blood pressure (BP) regulation, has been demonstrated to be responsible for the overactivity of the sympathetic nervous system in hypertension and heart failure. Nuclear factor-erythroid-2-related factor 2 (Nrf2) is a key transcription factor that maintains redox homeostasis by governing a broad array of antioxidant genes in response to oxidative stress. β-Arrestin1 is a multifunctional scaffold protein with the ability to interact with diverse signaling molecules independent of G protein-coupled receptors (GPCRs), and its overexpression in the RVLM could reduce BP and renal sympathetic nerve activity (RSNA) in spontaneously hypertensive rats (SHR). The goal of this study was to investigate whether Nrf2-mediated antioxidative stress is involved in the antihypertensive effect of β-arrestin1 in the RVLM. It was found that the activation level of Nrf2 in the RVLM of SHR was significantly reduced, compared with normotensive Wistar-Kyoko (WKY) rats. Overexpression of β-arrestin1 in the RVLM significantly decreased ROS production and facilitated the Nrf2 activation in the RVLM of SHR, accompanied by upregulating the expression of HO-1 and NQO-1. However, Nrf2 knockdown attenuated the antioxidant effect of β-arrestin1 overexpression in the RVLM by downregulating HO-1 and NQO-1 expression levels. In conclusion, the current results suggested that the antihypertensive effect of β-arrestin1 overexpression in the RVLM is mediated by decreased ROS production, which is associated with Nrf2 activation.

## Introduction

Oxidative stress occurs when the balance between the oxidation and antioxidant systems is broken, which results in the accumulation of reactive oxygen species (ROS) (Sack et al., 2017). Oxidative stress in the rostral ventrolateral medulla (RVLM), a crucial region for blood pressure (BP) and sympathetic regulation (Guyenet, 2006), is proved to be responsible for the overactivity of the sympathetic nervous system in animal models of hypertension (Wu et al., 2012), chronic heart failure (CHF) (Koba et al., 2014), and traumatic brain injury (Chen et al., 2019). Numerous studies have suggested that compared with normotensive Wistar-Kyoko (WKY) rats, the ROS production of RVLM in spontaneously hypertensive rats (SHR) is markedly enhanced, and overexpression of superoxide dismutase (SOD) in the RVLM of SHR decreases the levels of BP and renal sympathetic nerve activity (RSNA) (Chan et al., 2006, 2009). RVLM microinjection of SOD mimic Tempol significantly reduces the BP and heart rate (HR) of hypertensive rats (Kishi et al., 2004). It is indicated that overactivity of the sympathetic nervous system in hypertension is closely related to oxidative stress, and exploring the antioxidant strategy in the RVLM has great prospective in the treatment of hypertension.

β-Arrestin is a key regulatory protein of G protein-coupled receptor (GPCR), including β-arrestin1 and β-arrestin2. β-Arrestin plays a negative role in regulating GPCR by regulating desensitization and endocytosis and mediating cell signal transduction (Vaughan et al., 2006). Recent studies have found that β-arrestin exerts a cardiovascular protective effect by transactivating epidermal growth factor receptors in the heart (Noma et al., 2007). Moreover, it has been reported that β-arrestin1 exerts neuroprotective effects by regulating autophagy mediated by Beclin-1 in the cerebral ischemia model (Wang et al., 2014). Meanwhile, our previous study has demonstrated that β-arrestin1 overexpression in the RVLM could reduce BP and RSNA in SHR (Sun et al., 2018). Interestingly, it is indicated that β-arrestin1 is closely related to the regulation of oxidative stress. For instance, under the stimulation of the CXR2 receptor with IL8, β-arrestin can prevent the occurrence of oxidative stress-mediated cell death of mouse embryonic fibroblasts (Zhao et al., 2004). However, it is not clear whether oxidative stress is involved in the process of β-arrestin1 overexpression in the RVLM improving cardiovascular function. In addition, the underlying mechanism of β-arrestin1 to regulate oxidative stress in hypertension is not yet clear.

Recent studies have reported that nuclear factor-erythroid-2-related factor 2 (Nrf2), a key transcription factor, is involved in the regulation of the antioxidant system *in vivo* (Lopes et al., 2015; Barancik et al., 2016). It has been demonstrated that *in vitro* and *in vivo* phosphorylation of Nrf2 on Ser40 by protein kinase C facilitates the dissociation of Nrf2 from the Keap1 complex. Phosphorylated Nrf2 (p-Nrf2) is involved in the nuclear translocation and antioxidant response element (ARE) transactivation of Nrf2 (Huang et al., 2002; Numazawa et al., 2003). Accumulating evidence has shown that Nrf2 plays an indispensable role in the pathogenesis of cardiovascular diseases. For example, selective Nrf2 deletion in the RVLM downregulates antioxidant enzymes and evokes hypertension and sympathoexcitation in mice (Gao et al., 2017). It is reported that microinjection IL-1beta into the RVLM decreases the expressions of p-Nrf2, which regulates mitochondrial biogenesis and participates in the pathogenesis of hypertension induced by systemic inflammation (Wu et al., 2016). Conversely, upregulating Nrf2 selectively in the RVLM attenuates sympathoexcitation in CHF mice (Ma et al., 2019). Taken together, these results indicate that Nrf2 participates in the maintenance of BP and sympathetic nerve activity through redox regulation. Nevertheless, what roles β-arrestin1 plays in Nrf2 regulation in the RVLM still remains elusive.

Accordingly, the present study is designed to investigate whether the Nrf2 pathway contributes to reduce oxidative stress in the RVLM and mediate the antihypertensive effect of β-arrestin1. Two main aims in this study need to determine (1) if β-arrestin1 in the RVLM exerts antihypertensive effects by attenuating the level of oxidative stress and (2) if Nrf2 mediates the antihypertensive effects of β-arrestin1 by regulating oxidative stress.

## Materials and Methods

### Animals

A total of 70 male SHR and 40 male WKY rats were used in the experiment. The rats weighing 250–350 g were 16 weeks old and purchased from Vital River Laboratory Animal Technology Co. Ltd. (Beijing, China). All procedures obtained approval from the Institutional Animal Care and Use Committee of Naval Medical University, and all experimental operations involving the use of animals in the present study strictly complied with the requirements in the Guide for the Care and Use of Laboratory Animals published by the US National Institutes of Health. Experimental rats were kept in a 12-h light and 12-h dark animal room, and they were allowed access to food and water *ad libitum*.

### Experimental Overview

**FIGURE 1:**
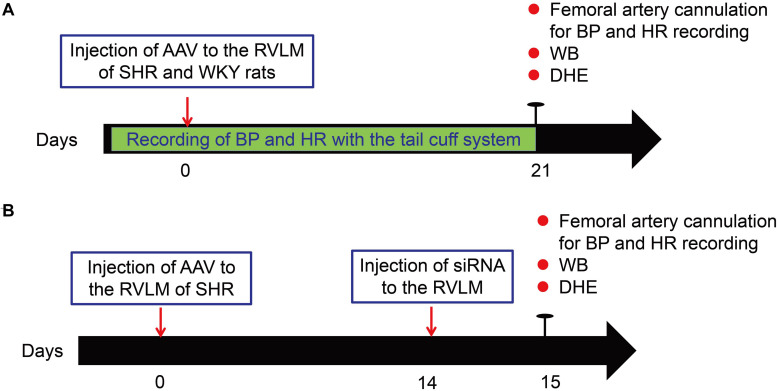
Experimental overview. **(A)** Schematic of general study design for the overexpression of β-arrestin1 in the RVLM of SHR and WKY rats. **(B)** Schematic of general study design for **β** -arrestin1 overexpression and Nrf2 knockdown in the RVLM of SHR. WB, Western blot; DHE, dihydroethidium.

Figure 1 shows the general study design. The SHR and WKY rats underwent BP and HR recording with the tail cuff system before adeno-associated virus (AAV) injection. After a 1-week acclimatization period, the rats were injected with AAV into the RVLM. Three weeks after injection, the BP and HR measurement of rats was performed though femoral artery cannulation. The brains of the rats after BP recording were removed and used for Western Blot and detection of ROS production (Figure 1A). For Nrf2 knockdown, on the 14th day after AAV injection, SHR rats were injected with small interfering RNA (siRNA) to evaluate the change of Nrf2 knockdown in the antioxidant stress effect of β-arrestin1 overexpression (Figure 1B).

### Construction of Overexpressing β-Arrestin1 Adeno-Associated Virus

In brief, as described previously (Sun et al., 2018), the overexpressing AAVs were constructed by OBiO Technology Corp., Ltd. (Shanghai, China). The overexpressing β-arrestin1 AAV carried the rat Arrb1 cDNA (Accession No. NM_012910) (AAV-Arrb1). The control AAV contained all sequences except the Arrb1 gene and was labeled by green fluorescence protein (AAV-GFP). The final infectious titer of AAVs for β-arrestin1 was 1.2 × 10^9^ transducing units/ml.

### Injection of AAV Into the RVLM

The rats were anesthetized by inhaling 3% isoflurane and fixed in the stereotaxic frame. As previously described (Sun et al., 2018), briefly, two holes were symmetrically drilled on the dorsal surface of the cranium after exposing the skull. According to the atlas of rats (Paxinos and Watson, 1998), the injection site for RVLM was determined as follows: 2.0 mm lateral to the midline, 3.5 mm caudal to the lambda, and 9.5–10 mm deep to the surface of the skull. AAV-Arrb1 or AAV-GFP particles (1 μl per side) were slowly bilaterally injected into the RVLM with a Hamilton syringe (5 μl) within 10 min. After completion of injection, incision was sutured. A thousand units of penicillin per kilogram of body weight was injected into the quadriceps muscle of rats to prevent infection.

### *In vivo* Nrf2 Knockdown

The siRNA that specifically interferes with the expression of Nrf2 protein was designed and synthesized by Shanghai Gene Pharmaceutical Co., Ltd. (Shanghai, China). The sequence of the specific siRNA against Nrf2 was as follows: sense (5′–3′) UUUGAGUCUAAGGAGUUCAGCUGGC, antisense (5′–′3) GCCAGCUGAACUCCUUAGACUCAAA, and the scramble sequence was used as the negative control (NC). As described previously (Xu et al., 2019), the transfection reagent Entranster^TM^-*in vivo* kit was purchased from Engreen Biosystem Co, Ltd (Beijing, China). According to the operation manual, the transfection complex was composed of siRNA, Entranster^TM^-*in vivo*, and DEPC water according to the ratio of 2:1:1, and the final concentration of siRNA in the transfection complex was 500 μM. To verify the efficiency of Nrf2 knockdown, the Nrf2 siRNA transfection complex (1 μl) was injected bilateral into the RVLM of WKY rats, and the rats were sacrificed at 24, 48, and 72 h after injection. Nrf2 protein expression was detected by Western blot to determine the most appropriate time for Nrf2 knockdown. Two weeks after overexpression of β-arrestin1 in the RVLM, SHRs were anesthetized and 1 μl transfection complex was microinjected bilaterally into the RVLM. The rat was anesthetized, and the brain was quickly removed 24 h later. SHRs were divided into four groups as follows, for different treatments: GFP-NC, GFP-Nrf2 siRNA, Arrb1-NC, and Arrb1-Nrf2 siRNA.

### Measurements of BP and HR

As described in our previous study (Wang et al., 2016), the BP and HR of conscious rats were monitored continuously by a non-invasive tail-cuff system (ALC-NIBP, Shanghai Alcott Biotech) before and after the injection of AAV-GFP and AAV-Arrb1 into the RVLM. For tail-cuff measurement, in order to avoid stress-induced fluctuations in BP of rats, the rats were allowed to adapt to the chamber and tail cuff for a period of 15–20 min before recording the BP. During this process, the rats rested comfortably in the chamber. The cuff was connected to the transducer, and the signal was amplified and recorded via the data acquisition system. All values of BP and HR were taken from the average of at least six consecutive periods.

Before the rats were sacrificed, the BP and HR in the anesthetized rats (urethane 800 mg/kg and α-chloralose 40 mg/kg, ip) were recorded through femoral artery cannulation. In brief, the trachea of anesthetized rats was cannulated to facilitate respiration with a ventilator (SAR-830, CWE). The end-tidal carbon dioxide (CO_2_) concentration was monitored by a CO_2_ analyzer (CapStar 100, CWE) and kept at the level of ∼4%. The right femoral artery was catheterized for BP and HR recording with the PowerLab system (8SP, AD Instruments). MAP and HR were obtained from the BP pulse.

### Detection of ROS Production in the RVLM

In the present study, the ROS production in the RVLM was detected by dihydroethidium (DHE), a ROS-sensitive fluorescent dye. DHE was dehydrogenated by ROS to produce ethidium. Ethidium binds to RNA or DNA to produce a red fluorescence. According to our previous study (Hao et al., 2016), the brains of the rats after BP recording were removed after being perfused with 0.09% saline throughout the body, quickly frozen with liquid nitrogen, and then sliced into 20 μm sections on a sliding freezing microtome. The brain sections were incubated with DHE (5 μmol/L) at 37°C for 30min and then washed in 0.1MPBS (3 × 5min). The sections were placed on a glass slide and were covered by a coverslip. The red fluorescence on the section was examined by a confocal laser scanning microscope (TCS-SP5, Leica, Germany), and the selected excitation wavelength was 530 nm. These images were evaluated using LAS-AF-Lite software.

### Western Blot

According to our previous study (Sun et al., 2018), rats were euthanized with an overdose anesthetic (urethane 2.4 g/kg and α-chloralose 120 mg/kg, ip). The brain was quickly removed, frozen with liquid nitrogen, and stored at −80°C. The RVLM tissues were punched and lysed in cell lysate for 10 min at 4°C. The samples were sonicated and centrifuged. Most of the supernatants were used for protein denaturation with loading buffer and heated to 100°C for 10 min; 1–2 μl of supernatant was used to determine the protein concentration using the BCA kit. After denaturation, the protein samples (30 μg) were loaded onto a 10% SDS-PAGE gel followed by transfer to the PVDF membrane. After blocking for 2 h at room temperature with 5% milk dissolved in Tris-buffered saline Tween (TBST), the membrane was incubated with primary antibodies [anti-keap1 (no. 8047, CST); anti-Nrf2 (no. ab92946, Abcam); anti-p-Nrf2 (no. orb506122, Biobyt); anti-β-arrestin1 (no. 32099, Abcam); anti-HO-1 (no. ab68477, abcam); anti-NQO-1 (no. ab80588, Abcam); and anti-α-tubulin (no. T6074, Sigma-Aldrich)] overnight at 4°C. The membranes were washed three times with TBST and then incubated with horseradish peroxidase-conjugated secondary antibodies for 2 h at room temperature. Finally, the target protein bands were exposed with chemiluminescent agent (Millipore) and analyzed by GeneTools software (Gene Company). The ratio of the target protein to α-tubulin was used to reflect the expression level of the target protein.

### Green Fluorescence Protein Fluorescence Detection

The protocols of immunofluorescence were described previously (Sun et al., 2018). In brief, the anesthetized rats were perfused with 0.9% saline and fixed with 4% paraformaldehyde in PBS. Then, the brain was removed and stored in 4% paraformaldehyde. Before frozen sectioning, the brains were dehydrated with 20% sucrose solution for 12 h. After being swift frozen, the brains were sliced into thin sections (10 μm), which were then mounted on slides and coverslipped with antifade medium. In addition, the GFP fluorescence was detected by laser confocal microscopy (TCS-SP5, Leica, Germany).

### Statistical Analysis

All data were examined as mean ± SE. When only two groups of data, SHR and WKY, were compared, the unpaired Student’s *t*-test was used. BP is expressed by mean arterial pressure. The BP monitored continuously on conscious rats were analyzed by repeated-measures ANOVA. In the Nrf2 siRNA knockdown efficiency test experiment, the expression difference of Nrf2 was analyzed by one-way ANOVA followed by Bonferroni’s *post hoc* test. All the other value differences were analyzed by two-way ANOVA followed by Turkey’s *post hoc* test. All the data were analyzed by GraphPad Prism 6.0 (GraphPad Software, San Diego, CA, United States). *P*-values of <0.05 were considered statistically significant.

## Results

### The Antihypertensive Effect of Overexpression of β-Arrestin1 in the RVLM

**FIGURE 2:**
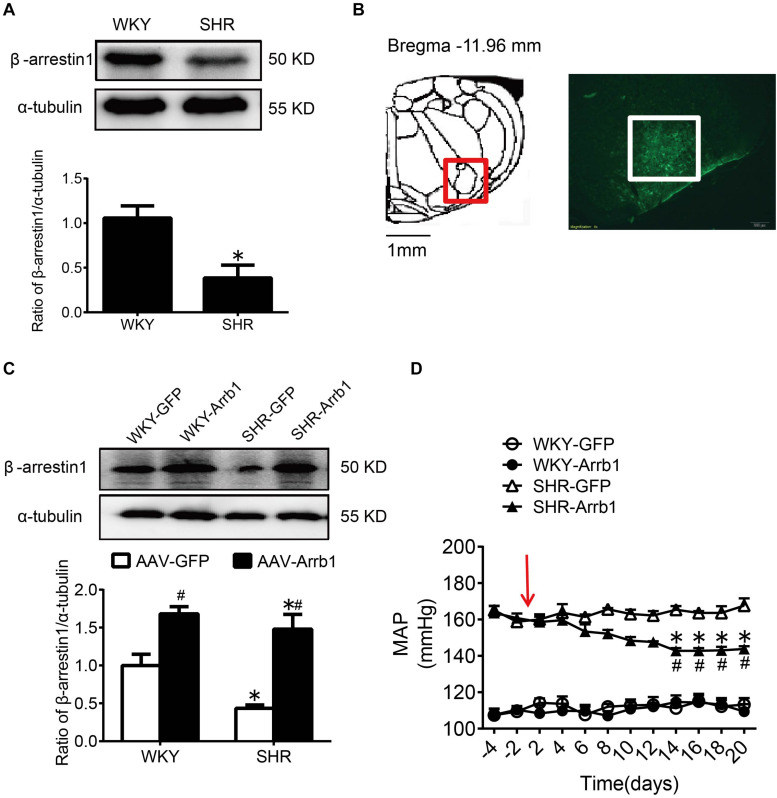
The antihypertensive effect of β-arrestin1 overexpression in the RVLM. **(A)** Western blot bands and statistical histogram for the β-arrestin1 expression in the RVLM of WKY rats and SHR. *P < 0.05 vs. WKY rats; *n* = 4/group. **(B)** The anatomical diagram of RVLM. GFP expression (green fluorescence) detection in the RVLM of WKY rats 14 days after injection of AAV-GFP particles. Scale bars = 200 μm. **(C)** Western blot bands and statistical histogram for the β-arrestin1 expression in the RVLM of WKY rats and SHR. *P < 0.05 vs. WKY rats; ^#^*P* < 0.05 vs. AAV-GFP; *n* = 5/group. **(D)** The level of mean arterial pressure (MAP) before and after adenovirus-associated virus transfection in conscious rats. The red arrow represents the injection of AAV. *P < 0.05 vs. AAV-GFP; ^#^*P* < 0.05 vs. BP before transfection; *n* = 5/group.

Consistent with our previous results (Sun et al., 2018), compared with WKY rats, the expression of β-arrestin1 in the RVLM was significantly decreased in SHR (Figure 2A). After ensuring that β-arrestin1 was successfully overexpressed in the RVLM (Figures 2B,C), compared with the AAV-GFP group, the BP of the AAV-Arrb1 group in SHR was decreased gradually and it maintained at a low level after 2 weeks of transfection. The BP between AAV-GFP and AAV-Arrb1 groups in WKY rats had no significant difference (Figure 2D).

### Overexpression of β-Arrestin1 in the RVLM Attenuated Oxidative Stress on SHR

**FIGURE 3:**
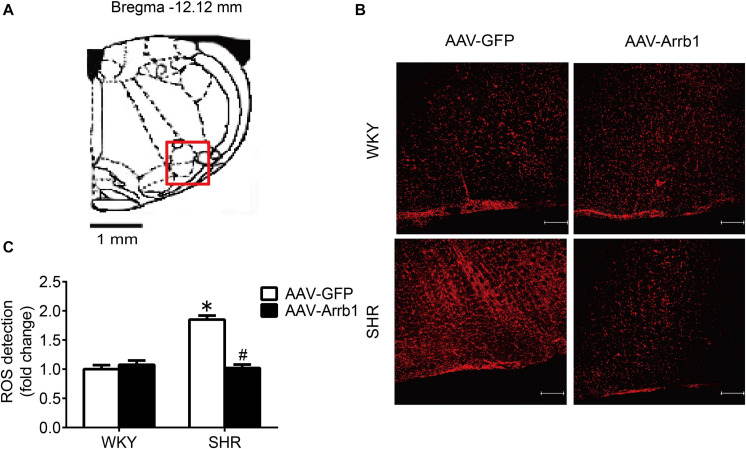
Overexpression of β-arrestin1 attenuated oxidative stress in the RVLM of SHR. **(A)** The standard atlas of RVLM in rats. **(B)** The fluorescence images of ROS detection in the RVLM. Scale bars = 200 μm. **(C)** The statistical histogram of ROS production in the RVLM in each group. *P < 0.05 vs. WKY rats; ^#^*P* < 0.05 vs. AAV-GFP; *n* = 5/group.

As shown in Figure 3, the ROS production in the RVLM of SHR was significantly higher than that in WKY rats in the AAV-GFP-treated groups. Compared with the AAV-GFP group, ROS production in the RVLM of the AAV-Arrb1 group was markedly attenuated in SHR. In contrast, the ROS production in the RVLM of the AAV-GFP and AAV-Arrb1 groups in WKY rats had no significant difference.

### The Overexpression of β-Arrestin1 Exerted an Antioxidant Stress Effect by Facilitating the Nrf2 Activation

**FIGURE 4:**
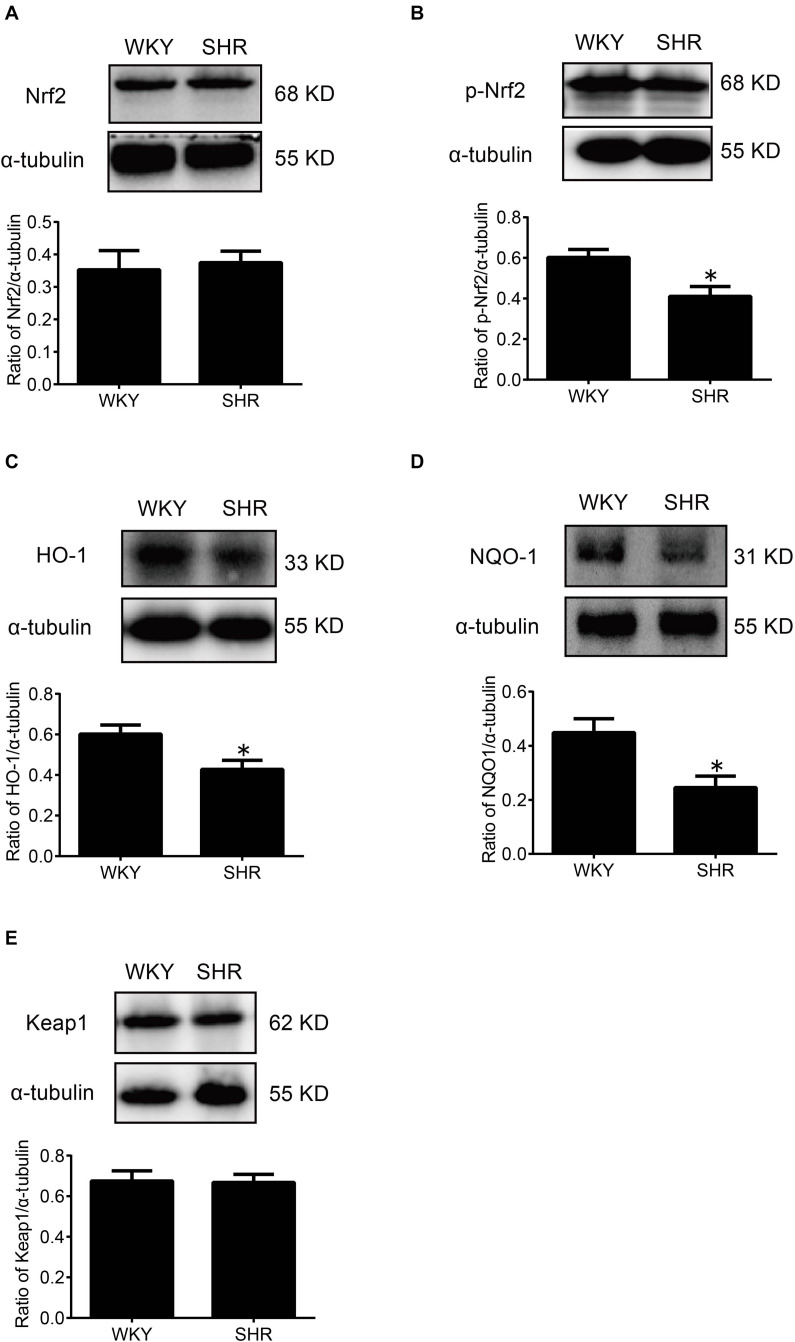
The activation levels of Nrf2 in the RVLM of WKY rats and SHR. **(A–E)** Western blot bands and statistical histograms for Nrf2, p-Nrf2, HO-1, NQO-1, and Keap1. *P < 0.05 vs. WKY rats; *n* = 5/group.

At first, the expression of p-Nrf2 in the RVLM was significantly decreased in SHR than that in WKY rats. Meanwhile, the expressions of HO-1 and NQO-1, both downstream antioxidant proteins of p-Nrf2, were also significantly reduced in SHR compared with WKY rats. However, the expression levels of Nrf2 and Keap1 were not changed significantly (Figure 4).

**FIGURE 5:**
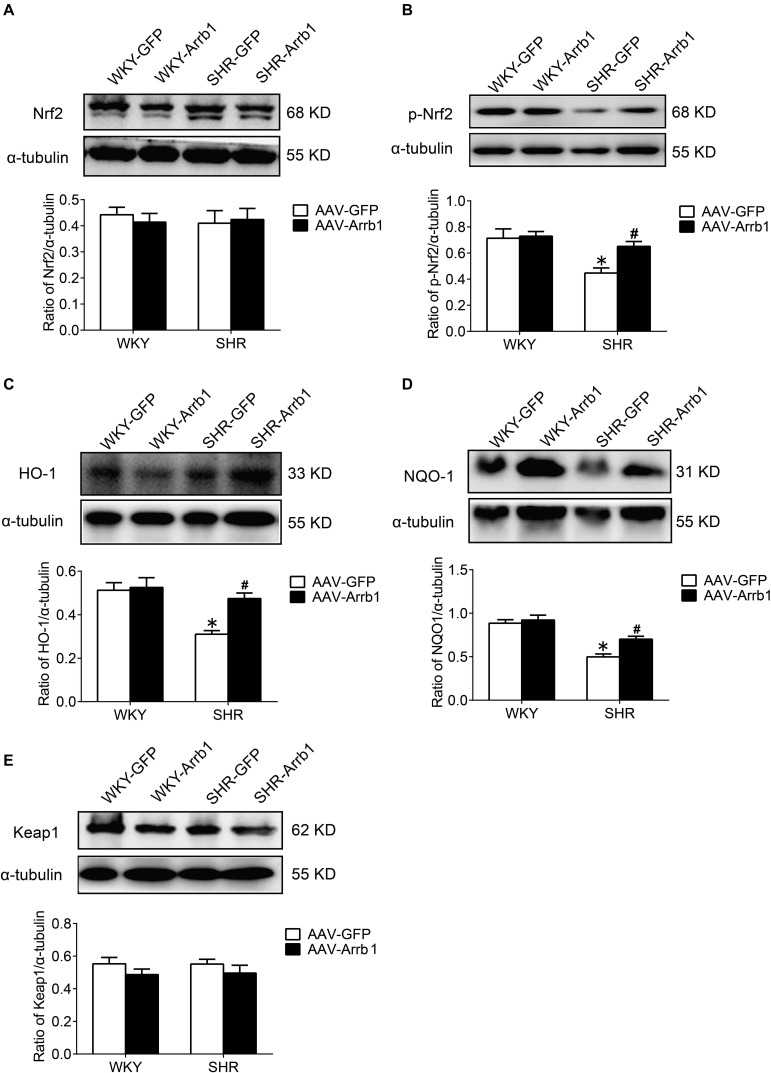
The effect of β-arrestin1 overexpression on the activation of Nrf2 and associated protein expression after in the RVLM of WKY rats and SHR. **(A–E)** Western blot bands and statistical histograms for Nrf2, p-Nrf2, HO-1, NQO-1, and Keap1 in the RVLM. *P < 0.05 vs. WKY rats; ^#^*P* < 0.05 vs. AAV-GFP; *n* = 4/group.

After overexpression of β-arrestin1, the level of p-Nrf2 expression in the RVLM of the AAV-Arrb1 group was markedly increased compared with the AAV-GFP group in SHR, accompanied by upregulation of the expression of HO-1 and NQO-1 (Figure 5), while the expression level of keap 1 did not differ significantly between the groups.

### Nrf2 Knockdown Reversed the Antioxidant Stress Effect of β-Arrestin1 Overexpression

**FIGURE 6:**
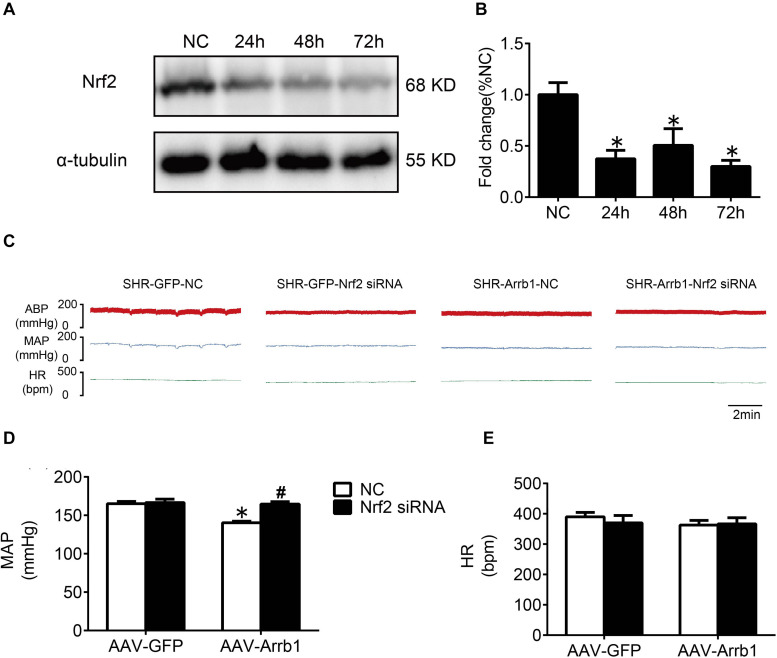
The efficiency and effect of Nrf2 knockdown in the RVLM. **(A,B)** Western blot bands and statistical histogram for Nrf2 expression in the RVLM at different times after injection of Nrf2-specific small interfering RNA in WKY rats. *P < 0.05 vs. NC group; *n* = 4/group. **(C–E)** The original graphs and statistical analysis graphs of BP and HR in SHR with different treatments under anesthesia. *P < 0.05 vs. AAV-GFP; ^#^*P* < 0.05 vs. NC; *n* = 4/group.

**FIGURE 7:**
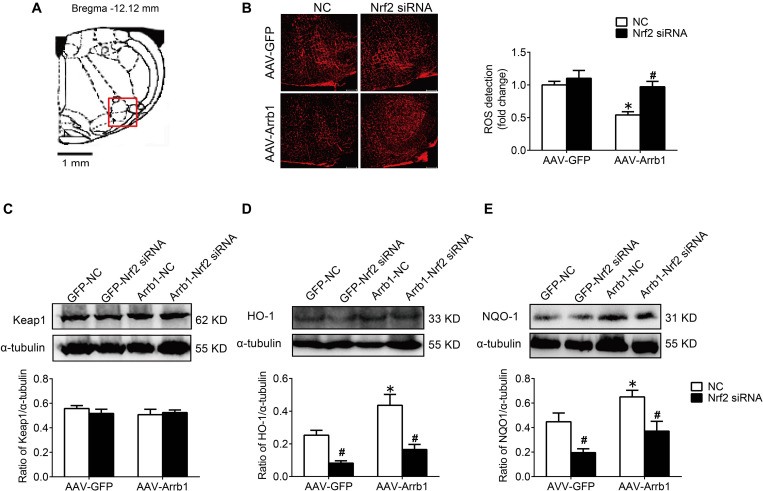
Nrf2 knockdown reversed the antioxidant stress effect of β-arrestin1 overexpression in the RVLM of SHR. **(A)** The RVLM (red frame) in standard atlas of rats. **(B)** The fluorescence images and statistical histogram of ROS detection in the RVLM. Scale bars = 200 μm. **(C–E)** Western blot bands and statistical histograms of Keap1, HO-1, and NQO-1 expression in the RVLM in groups. *P < 0.05 vs. AAV-GFP; ^#^*P* < 0.05 vs. NC; *n* = 5/group.

In order to verify the efficiency of Nrf2 knockdown, Western blot was used to detect the protein expressions of Nrf2 at 24, 48, and 72 h after Nrf2 siRNA injection into the RVLM. Compared with the NC group, the protein expressions of Nrf2 at 24, 48, and 72 h after siRNA injection were significantly reduced, and there was no significant difference between the three groups (Figures 6A,B). Therefore, we chose to observe the effect at 24 h after the injection of Nrf2 siRNA in the follow-up experiments. The BP of the SHR-Arrb1-Nrf2 siRNA group was markedly higher than that in the SHR-Arrb1-NC group, and the BP between the SHR-GFP-Nrf2 siRNA group and SHR-GFP-NC group had no significant difference, whereas there was no significant difference in HR between the groups (Figures 6C–E). Furthermore, the ROS production in the RVLM of the SHR-Arrb1-Nrf2 siRNA group was markedly increased than that in the SHR-Arrb1-NC group. However, there was no difference between the SHR-GFP-Nrf2 siRNA group and SHR-GFP-NC group (Figures 7A,B). Compared with the SHR-Arrb1-NC group, the expression levels of HO-1 and NQO-1 in the RVLM of the SHR-Arrb1-Nrf2 siRNA group were obviously downregulated. Nevertheless, there was no significant difference in the level of Keap1 expression between the groups (Figures 7C–E).

## Discussion

The present study reveals the effect of β-arrestin1 on oxidative stress and confirms that β-arrestin1 exerts an antihypertensive effect mediated by Nrf2 activation. In this study, the major findings are that (1) overexpression of β-arrestin1 improved the cardiovascular dysfunction in SHR by decreasing the level of ROS in the RVLM and (2) overexpression of β-arrestin1 facilitated the Nrf2 activation in the RVLM of SHR, while Nrf2 knockdown reversed the antioxidant stress effect of overexpression of β-arrestin1. Based on our findings, it is indicated that the overexpression of β-arrestin1 decreases the BP as a result of its antioxidant effect mediated by facilitating Nrf2 in hypertension.

β-Arrestin1 in the RVLM is involved in the neural regulation of BP and has the effect of improving the cardiovascular function of SHR, but its detailed mechanism is still unclear. Our previous studies have confirmed that overexpression of β-arrestin1 in the RVLM reduces BP and sympathetic outflow in hypertension by downregulating the expression of angiotensin type I receptors (AT1R) and revealed that this effect is mediated through the enhancement of interaction β-arrestin1 and IκB-α (Sun et al., 2018). Conversely, one study has reported that β-arrestin overexpression in normal cardiac fibroblasts increases mitochondrial superoxide production twofold, which are prevented by inhibition of either NADPH oxidase 4 or extracellular signal-regulated kinase (ERK) (Philip et al., 2015). This conflicting result indicates that the effect of overexpression of β-arrestin may be different *in vivo* and *in vitro*. Combining with the results of our previous and current *in vivo* experiments, it is suggested that overexpression of β-arrestin1 in the RVLM exerts antihypertensive effects, which is mediated not only by reducing the expression of AT1R but also by reducing oxidative stress. These findings suggest that β-arrestin1 in the RVLM is involved in the neural regulation of cardiovascular function through multiple mechanisms.

A large amount of research evidence has shown that increased levels of oxidative stress in the RVLM contributes to sympathetic output and increases BP, and abnormal enhancement of the renin–angiotensin system (RAS) in the RVLM promotes oxidative stress (Griendling and FitzGerald, 2003; Malpas, 2010). Our previous work has found that overexpression of β-arrestin1 in the RVLM reduces the expression of AT1R and inhibits RAS (Sun et al., 2018). Although this study did not design future experiments to test whether overexpression of β-arrestin1 indirectly reduces the level of oxidative stress in the RVLM by downregulating AT1R, we further explored the pathways involved in the antioxidant effects of β-arrestin1 overexpression and found that overexpression of β-arrestin1 in the RVLM significantly enhanced Nrf2 activation and decreased the ROS production, all of which were abrogated by treatment with Nrf2 siRNA. Therefore, we conclude that the overexpression of arrestin1 in the RVLM exerts an antihypertensive effect by activating Nrf2 to reduce the level of oxidative stress.

Excessive generation of ROS is an important aspect of oxidative stress. The increased activities of NADPH oxidase, myeloperoxidase, and cytochrome P450 monooxygenase; nitric oxide synthase uncoupling; and mitochondrial respiratory chain dysfunction contribute to the ROS production (Brandes et al., 2010). On the other hand, abnormalities in the ROS clearance mechanism also result in oxidative stress (Sack et al., 2017). In addition to the above mechanisms, studies have found that Nrf2 plays an important role in the defense against oxidative stress and the maintenance of redox homeostasis in recent years (Barancik et al., 2016). Under the physiological condition, Nrf2 binds to Keap1 protein in an ubiquitinated manner and exists in the cell cytoplasm. When the cell is subjected to external stimuli such as ROS, the cysteine residue of Keap1 is oxidized, and its conformation changes and depolymerizes with Nrf2 and is phosphorylated to form p-Nrf2. After activation, Nrf2 in the cytoplasm enters the nucleus and combines with ARE to initiate an endogenous antioxidant response, for instance, promoting the expression of HO-1 and NQO-1 (Barancik et al., 2016; Satta et al., 2017). This study found that the level of Nrf2 downstream proteins HO-1 and NQO-1 in the RVLM of SHR was significantly reduced, and overexpression of β-arrestin1 in the RVLM significantly upregulated the expression of HO-1 and NQO-1, all of which were abrogated by Nrf2 knockdown. However, the level of Keap1 expression was not significantly different between different treatment groups. These findings suggest that the increased level of oxidative stress in the RVLM is closely related to the decreased activation of Nrf2, and it is achieved by downregulating the expression of HO-1 and NQO-1 instead of Keap1.

In the light of the research results of our and other teams (Gao et al., 2017; Ma et al., 2019), knockdown of Nrf2 in the RVLM leads to an increase in ROS production and increase in sympathetic nerve activity and BP. It is indicated that the reduction of Nrf2 activation is one of the important mechanisms for the enhancement of oxidative stress in the RVLM involved in the pathogenesis of hypertension. We have shown that the expression levels of Nrf2 and p-Nrf2 were decreased in SHR treated with AAV-GFP-Nrf2 siRNA compared with SHR treated with AAV-GFP-NC (Supplementary Figure 2). However, the ROS production and MAP were not altered in SHR treated with AAV-GFP-Nrf2 siRNA compared with SHR treated with AAV-GFP-NC. The possible reason for these results is that Dr. Gao’s team knocked down Nrf2 in mice with normal BP, whereas knockdown of Nrf2 was performed in SHR in this study. The expression level of Nrf2 in RVLM of SHR was lower than that in WKY rats, and the expression level of Nrf2 in the RVLM of SHR was already at a relatively low level. If Nrf2 was further knocked down, the effect of increasing ROS production and BP was not as obvious as that of animals with normal BP; even ROS production and MAP were not altered after knockdown Nrf2 in SHR treated with AAV-GFP. On the other hand, there is evidence that Nrf2 has a dual role in cardiovascular disease. Autophagy impairment leads to the accumulation of Nrf2 in the nucleus, which increases the expression of angiotensinogen, thereby increasing the level of Angiotensin II, resulting in cardiac dysfunction (Qin et al., 2016). However, the mechanism of regulation of Nrf2 activation under hypertension is not clear. It was reported that resveratrol, a polyphenolic stilbene, restores Nrf2 expression and ameliorates inflammation and oxidative stress, which attenuates severity and progression of hypertension in SHR (Javkhedkar et al., 2015). At the same time, it has been confirmed that the phosphorylation of Nrf2 is decreased in the vascular smooth muscle cells of hypertensive rats, while L-sulforaphane, an Nrf2 agonist, attenuates the vascular dysfunction of hypertensive rats (Lopes et al., 2015). Additionally, exercise training upregulates Nrf2 protein in the RVLM of heart failure mice (Wafi et al., 2019). Our research results confirmed that overexpression of β-arrestin1 facilitated the Nrf2 activation, which provides a new strategy for the regulation of Nrf2.

Although the present study confirms the relationship between β-arrestin1’s anti-hypertension and oxidative stress, there are also some limitations. First, this study has not carried out further exploratory experiments on the specific pathway through which β-arrestin1 promotes the activation of Nrf2. It has been reported that thrombin can activate the PI3K/Akt signaling pathway mediated by Gα and β-arrestin1 in IIC9 cells (Goel and Baldassare, 2002). Under the condition that HEK293 cells are stimulated by angiotensin II, β-arrestin plays a scaffold-like effect to promote ERK activation (Ahn et al., 2004). Furthermore, it was confirmed that α-lipoic acid reduces the area of myocardial infarction by activating the PI3K/Akt/Nrf2 signaling pathway, resulting in protective effects of cardiovascular function in the myocardial ischemia–reperfusion rats (Deng et al., 2013). It has been reported that high glucose downregulates the expression of Nrf2 by activating the ERK signaling pathway to promote the production of oxidative stress in the cardiomyocytes of diabetic mice (Tan et al., 2011). Based on the above research evidence, we speculate that β-arrestin1 may activate Nrf2 through PI3K/Akt or ERK signaling pathways, thereby exerting its antioxidative stress effect. However, combined with the results of our previous study (Tan et al., 2017), the PI3K/Akt pathway in the RVLM of hypertensive rats was activated, and inhibition of the PI3K/Akt pathway ameliorated neuroinflammation, thereby reducing BP. Given that the PI3K/Akt pathway mediates neuroinflammation and promotes the occurrence of hypertension, overexpression of β-arrestin1 exerts an antihypertensive effect through Nrf2 activation. Therefore, in the pathway selection of β-arrestin1 which promotes the activation of Nrf2, we prefer the ERK signaling pathway. In future research, we will focus on this pathway.

**FIGURE 8:**
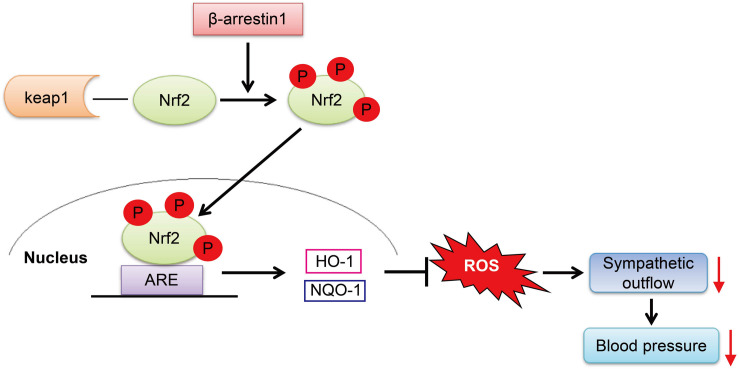
The working model of the β-arrestin1 overexpression reduces BP. β-Arrestin1 overexpression activates Nrf2, upregulates the expression of antioxidant enzymes such as HO-1 and NQO-1, attenuates oxidative stress, and results in a decrease in sympathetic output and BP.

In conclusion, we have demonstrated that the overexpression of β-arrestin1 in the RVLM exerts an antihypertensive effect mediated by a decrease in ROS production, which is associated with Nrf2 activation (Figure 8). These findings provide a new theoretical basis for the cardiovascular protective effect of β-arrestin1 in the RVLM and a new strategy for improving the oxidative stress of hypertension.

## Data Availability Statement

The datasets presented in this study can be found in online repositories. The names of the repository/repositories and accession number(s) can be found in the article/Supplementary Material.

## Ethics Statement

The animal study was reviewed and approved by the Institutional Animal Care and Use Committee of Naval Medical University.

## Author Contributions

XT, P-LJ, and J-CS contributed to conduct the experiments. WW, PY, and Y-QL collected and analyzed the data of the study. XT drafted the initial version of the manuscript. Y-KW and W-ZW provided revisions on the manuscript. All authors have read and approved the final version of the manuscript for publication.

## Conflict of Interest

The authors declare that the research was conducted in the absence of any commercial or financial relationships that could be construed as a potential conflict of interest.
