# Supplementary material for: β-Arrestin1 Reduces Oxidative Stress via Nrf2 Activation in the Rostral Ventrolateral Medulla in Hypertension
Source: Front Neurosci. 2021 Apr 7;15:657825. doi: 10.3389/fnins.2021.657825 (PMC8059792; doi:10.3389/fnins.2021.657825)

Figure legends

Supplementary Figure 1. The β-arrestin1 was expressed in the in the NTS and PVN. (A) Western blot bands and statistical histogram of β-arrestin1 expression in the NTS of WKY rats and SHR. (B) Western blot bands and statistical histogram of β-arrestin1 expression in the PVN of WKY rats and SHR. *P<0.05 vs. WKY rats; n=4/group.

Supplementary Figure 2. The effect of Nrf2 knockdown on the expression of Nrf2 and p-Nrf2 in the RVLM of SHR. (A) Western blot bands and statistical histograms of Nrf2 expression in the RVLM in groups. (B) Western blot bands and statistical histograms of p-Nrf2 expression in the RVLM in groups. *P<0.05 vs. AAV-GFP; #P<0.05 vs. NC; n=5/group.

Supplementary Figure 1

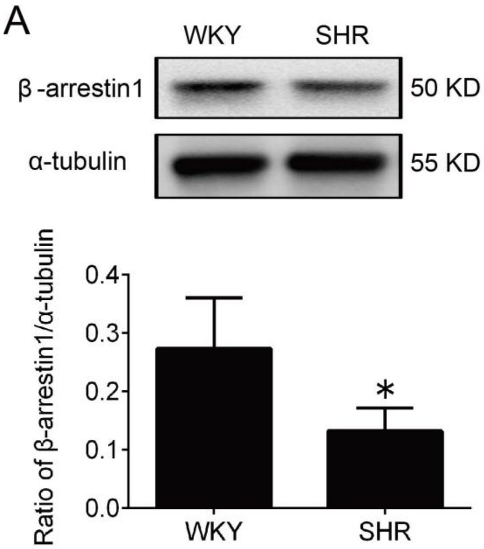

Supplementary Figure 2

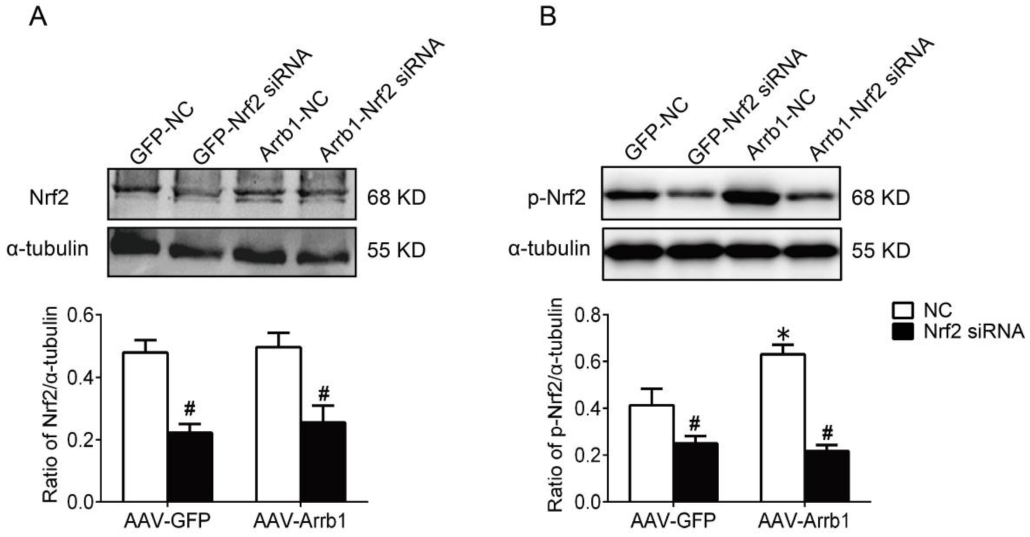

Supplement: Supplementary file 1 [file Data_Sheet_1.PDF]
